# The Role of Hydrogel in Cardiac Repair and Regeneration for Myocardial Infarction: Recent Advances and Future Perspectives

**DOI:** 10.3390/bioengineering10020165

**Published:** 2023-01-27

**Authors:** Ping Li, Jiajia Hu, Jian Wang, Junjie Zhang, Lu Wang, Chengliang Zhang

**Affiliations:** 1 Department of Obstetrics, Xiangya Hospital, Central South University, Changsha 410008, China; 2 Department of Anesthesiology, Xiangya Hospital, Central South University, Changsha 410008, China; 3 Department of Cardiovascular Surgery, Xiangya Hospital, Central South University, Changsha 410008, China; 4 National Clinical Research Center for Geriatric Disorders, Xiangya Hospital, Central South University, Changsha 410008, China

**Keywords:** hydrogel, cardiac repair, cardiac regeneration, myocardial infarction

## Abstract

A myocardial infarction (MI) is the leading cause of morbidity and mortality, seriously threatens human health, and becomes a major health burden of our society. It is urgent to pursue effective therapeutic strategies for the regeneration and restore myocardial function after MI. This review discusses the role of hydrogel in cardiac repair and regeneration for MI. Hydrogel-based cardiac patches and injectable hydrogels are the most commonly used applications in cardiac regeneration medicine. With injectable hydrogels, bioactive compounds and cells can be delivered in situ, promoting in situ repair and regeneration, while hydrogel-based cardiac patches reduce myocardial wall stress, which passively inhibits ventricular expansion. Hydrogel-based cardiac patches work as mechanically supportive biomaterials. In cardiac regeneration medicine, clinical trials and commercial products are limited. Biomaterials, biochemistry, and biological actives, such as intelligent hydrogels and hydrogel-based exosome patches, which may serve as an effective treatment for MI in the future, are still under development. Further investigation of clinical feasibility is warranted. We can anticipate hydrogels having immense translational potential for cardiac regeneration in the near future.

## 1. Introduction

Myocardial infarction (MI), which is caused by energy and oxygen demand blockage of the coronary artery, leads to massive amounts of cardiomyocytes death, exacerbated inflammatory response, pathological ventricle remodeling, disruption of electrical signaling, decreased systolic and diastolic function, eventually leading to heart failure (HF) [1,2]. MI kills around 7 million people worldwide per year. It is the leading cause of morbidity and mortality, and seriously threatens human health; it is becoming a major health burden for our society [3,4,5].

The current treatment approaches, including pharmacological treatment, percutaneous coronary intervention (PCI), and coronary artery bypass surgery (CABG), mainly focus on the restoration of blood supply to the ischemic cardiomyocytes [6]. No available drug can eliminate the loss of cardiomyocytes to erase the impairment caused by MIs [7]. In revascularization strategies, both CABG and PCI are unable to rescue the affected ischemic and arrest pathology progression of cardiac remodeling and subsequent HF due to the limited self-regeneration capability of the cardiomyocytes [8]. Meanwhile, ischemic reperfusion injury may incur in the condition when the blood supply is restored immediately after MI [9]. Moreover, the revascularization cannot reverse the adverse cardiac remodeling, which contributed to cardiomyocyte apoptosis, and cardiac fibrosis, ultimately inducing HF and even death [6]. These treatments focus on restoring the blood flow and are primarily unable to delay the progression of pathological cardiac remodeling without substantial benefit to cardiac regeneration. Research reported that therapeutic strategies that induce cardiac regeneration have been promising approaches for HF after MI [10]. It is urgent to pursue effective therapeutic strategies for the regeneration and restore myocardial function after MI.

## 2. Biomaterials in Cardiac Regeneration Medicine

Cell therapy and extracellular vesicle (EV)-based therapy have been extensively studied for tissue regeneration, and shown promising therapeutic potential in restoring cardiac function in MI. Cell therapy, which mainly focused on mesenchymal stem cells (MSCs) and cardiac stem cells (CSCs), suffers from low cell survival, engraftment and retention, and risk from immunogenicity and tumorgenicity [11]. EV-based therapy shows the potential for cardiac regeneration by promoting pro-regenerative and terminating fibrotic signaling pathways, which are mainly mediated by different cargos [12]. However, disadvantages including lower colonization resistance in the heart and high clearance from blood limit the application scenarios of EV-based therapy in MI.

**Figure 1:**
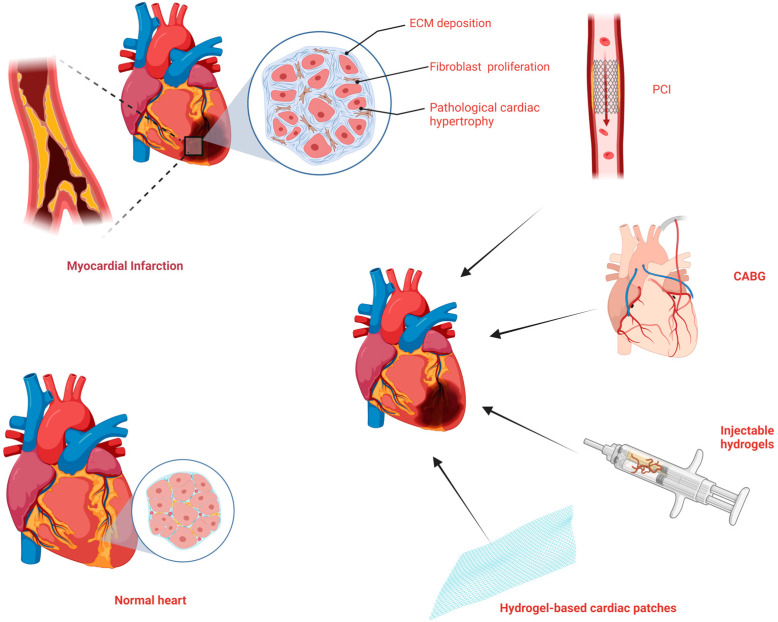
The pathophysiological changes and treatment of myocardial infarction. Created with BioRender.com.

Biomaterials can be used to encapsulate compounds, including cells or EVs, provide a suitable microenvironment for the survival of compounds, and show enormous potential in cardiac regeneration. Numerous advantages of biomaterials, including unlimited availability, stabilizability, unique bioactivities, and biocompatibility, arouse great interest in research regarding their potential for cardiac repair and regeneration (Figure 1) [13]. Additionally, the mechanical properties and bioactivities of biomaterials can be influenced by intrinsic and extrinsic stimuli, such as temperature, pH, hypoxia, reactive oxygen species (ROS), enzymes, light, ultrasound, magnetism, and electricity [14,15]. As one of the most attractive properties, the stimuli-responsive behavior expands the application scenarios.

Biomaterials have unique advantages over non-degradable materials, which harmfully influence the biocompatibility of tissue with immune activation due to the alloying elements [16,17]. As an emerging interdisciplinary science, tissue engineering combines the principles of biomedical engineering, material sciences, and life science, aiming to the regeneration of injured tissue to enhance or restore tissue functions [18,19]. Tissue engineering has transformed the field of regenerative medicine, shown enormous potential in cardiac tissue repair and regeneration overcoming the limitations of current treatments of MI, and entering a new era of heart repair and regeneration [20,21]. Biomaterials, including injectable hydrogels, cardiac patches, vascular grafts, and other nanocarriers have been studied extensively in cardiac regeneration medicine [22,23,24].

Various criteria have been proposed for the classification of biomaterials. Generally, biomaterials fell into three categories: organic, inorganic, and bio-based materials. Meanwhile, biomaterials are categorized as natural and synthetic materials based on their origins [25]. Biotherapy based on biomaterials has increasingly become a hotspot field with bright prospects in multiple areas, such as cancer immunotherapy, gene therapy, cell-based therapy, EV-based therapy, and tissue engineering [25].

## 3. The Property of Biomaterials for Cardiac Regeneration Medicine

The property of biomaterials, including mechanical properties, biocompatibility, biostability, biodegradability, unique bioactivities, and maneuverability influences the function of biomaterials in biomedical and clinical research [25]. As an important property, the biodegradability of biomaterials contributes to tissue repair and regeneration. Biomaterials should be degraded gradually at a reasonable rate without inflammatory reaction or toxicity during the period of tissue repair and regeneration, and not hinder the regeneration process. The physicochemical characteristics of biomaterials, such as size, shape, rigidity, surface charge, and contact angle, influence the efficacy of biomaterials [26]. Meanwhile, cardiovascular-specific properties of the heart should be considered in the development of biomaterials. The histologic components of the heart are cardiac muscle tissue, heart valves, and blood vessels, with the myocardium being the thickest muscular layer capable of enduring rapid, repeated contractions, relaxations, and electrical conductions of a heart [27].

It is essential to optimize biomaterials for the heart to mimic the natural heart’s specific characteristics, including heartbeat, electrical conductivity [28], and the bloodstream. Due to its poor elasticity, the material may not be able to withstand the mechanical dilation of the ventricular walls by cardiomyocytes. This may result in inadequate retention and growth capacity for the cells [29]. As a result of the limited conductivity, intercellular communication is hindered by a lack of signal conduction abilities. Cardiac tissue engineering should consider these cardiovascular properties, including elasticity, conductivity, and contractility of cardiomyocytes while seeking to enhance the survival and retention of cardiomyocytes and stem cells in situ [30,31]. Chronic heart failure can result in death within a short time, which assigns to the reduction in contractility and conductivity caused by inflammation after biomaterial patches application [32]. On-target bioactivity accumulation must be promoted while off-target and undesirable side effects need to be minimized. The bloodstream can wash away the materials from the pulsatile heart, and immunogenicity may hinder the formation of a suitable microenvironment for repair. The application of biomaterials, including the bioactive agent administration route and the surgical manipulation, should take into consideration. Moreover, the biocompatibility of biomaterials is essential to avoid immune rejection or cardiotoxicity [33], which is associated with the retention of cardiomyocytes and growth to colonize target organs [29]. Furthermore, an anticoagulant is noteworthy since there is a bloodstream in the blood vessels, and immune-mediated coagulopathy may incur after the application [34].

## 4. Hydrogel in Cardiac Repair and Regeneration for MI

Hydrogels are polymer-based materials with a three-dimensional network structure that attracts, contains, and becomes swollen with water [35,36]. There are several advantages to hydrogels, including a high binding affinity, variable pores, water absorption, biodegradability, and electrical conductivity [37]. It is expected that hydrogels will have exceptional potential in many applications including drug delivery carriers [38], sensors [39], disease treatment scaffolds [40], and wound dressings [41], as well as scaffolds for tissue engineering [42] and wearable devices [43].

Due to their biocompatibility and biomimetic nature, hydrogel-based biomaterials gained prior attention for cardiac regeneration. Since hydrogels fulfill most of these requirements, they serve as ideal biomaterials for cardiac implants and delivery systems. They have a cross-linked spatial structure, high biocompatibility, low immunogenicity, and can administer multiple bioactive agents into damaged organs or tissues [44,45]; they enhance the success rate of bioactive agents and prolong the half-life in vivo [44]; and they reduce the rapid degradation of protein molecules [46]. Moreover, hydrogels diminish the adverse side effects caused by insoluble bioactive compounds. Hydrogels’ characteristics can be stimulated by environmental changes that make an extensive range of applications [47]. Furthermore, hydrogel cardiac patches or bioactive agents can be delivered in a minimally invasive process [21,48].

As is well known, chemical and physical characteristics influence the properties of hydrogels. The rigidity and rheological parameters contribute to the mechanical stability of hydrogels, while the characteristics of the porosity, conductivity, contractibility, density, and stiffness impart the mechanical strength of hydrogels, which contributes to their potential applications. Natural biomaterials have several chemical and physical characteristics, such as structural similarity to natural tissue, which makes them bioactive, biodegradable, nonimmunogenic, and non-thrombogenic biomaterials. The high stiffness of natural biomaterial-based hydrogels provides sufficient mechanical strength. However, the limited composition consistency may incur immunogenicity events. The gelation time and degradation pattern also can be easily interfered with the low composition consistency. Synthetic biomaterial-based hydrogels show high consistency in their production, and great control over their production, which can allow changing their composition and physical features such as porosity, conductivity, contractibility, density, and stiffness in a reproducible way.

## 5. Intelligent Hydrogels and Cardiac Tissue Engineering

**Figure 2:**
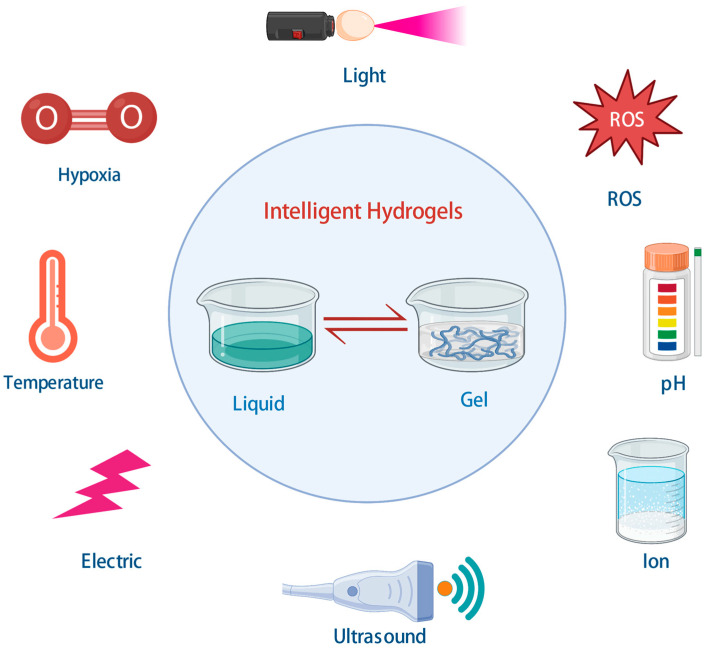
Intelligent hydrogels and cardiac tissue engineering. Created with BioRender.com.

Hydrogels as water-enriched polymeric biomaterials mimic the extracellular matrix of the heart. The polymeric backbone of hydrogels makes structural rearrangements by physiochemical alterations in the surrounding medium, transforming into stimuli-responsive hydrogels, which are called intelligent hydrogels. The base polymer of hydrogels with responsive function is designed in vivo, and the rearrangements react appropriately in response to stimuli in the target site [49]. The physiochemical structure rearrangements in response to intrinsic and extrinsic stimuli, such as temperature, pH, ion, hypoxia, and ROS (Figure 2) [14,15]. The applications of intelligent hydrogels in regenerative cardiology, including direct implantation onto the left ventricle with a controlled drug release system [50], optimize post-ischemic environments with angiogenic factors [51], provide structural support to the injury area, and enhance conductivity [52].

### 5.1. Temperature-Responsive Hydrogels

Temperature can change the conformations of hydrogels. Hydrogel undergoes a reversible transition from the soluble liquid to the insoluble gel phase at lower critical solution temperature, and transitions to hydrophobic from the hydrophilic phase above the temperature limitation [53].

Temperature-responsive hydrogels are mainly based on the amphiphilic polymer, poly(N-isopropylacrylamide) (PNIPAAm) [53], poly-(DL-lactic acid co-glycolic acid (PLGA-PEG-PLGA) [54], Pluronics^®^ F-127 [55] poly(N-vinylcaprolactam) (PVCL) [56], and poly(*N*,*N*-dimethylaminoethyl methacrylate methacrylate) (PDMAEMA) [57]. Due to their thermo-responsive properties, temperature-responsive hydrogels show promising applications in biomedicine, including wound healing, tumor treatment, tissue regeneration via cell delivery, and on-demand drug delivery [58,59].

Moreover, a minimally invasive delivery system is being developed for thermo-responsive hydrogels [60,61,62,63]. Under physiologic conditions (37 °C), these hydrogels allow components to be injected into the liquid phase via a catheter, which solidifies into a gel. As a result, localized injections can provide mechanical support for cardiac muscles in the event of an MI, for example [64]. Cardiovascular remodeling is ameliorated and cardiac regeneration is accelerated using targeted thermo-responsive hydrogel therapy in conjunction with pro-angiogenic drugs.

Using a temperature-responsive injectable polymer-mediated delivery system for adipose-derived stem cells, the cells can remain at the injection site longer and recover more quickly from ischemia [65]. With a pH- and temperature-sensitive hydrogel, continuous and localized cytokine release in MI rats was induced by pH and temperature, resulting in accelerated angiogenesis, the proliferation of cardiomyocytes, the inhibition of myocardial fibrosis, and improved cardiac function [66]. During cardiomyocyte transplantation using temperature-responsive hydrogels, infarct size was dramatically reduced, transplanted cardiomyocytes were better integrated, vascularization in the infarct zone was stimulated, and cardiac function was substantially improved [67].

### 5.2. pH-Responsive Hydrogel

A pH-sensitive hydrogel is made up of a polymer backbone with an acid or basic group that hydrolyzes at an accelerated rate in acidic conditions [68]. When polymeric backbones are exposed to basic environments, carboxylic acid moieties in the polymer backbone ionize, transitioning from gel to liquid hydrogel [69]. The pH-responsive property is advantageous for tissue engineering and sustained drug delivery, and has demonstrated promise in cancer, infection, and ischemia pathologies [70]. Research reported that pH-responsive, injectable base hydrogels with improved solubility and stability show promise as drug carriers that selectively target cancer cells [71]. A lower pH is observed in infarcted myocardia compared to healthy cardiac tissue [72], suggesting pH can be used as a heart-targeted delivery stimulus in ischemic myocardium, as well as opening up opportunities to design pH-responsive hydrogel delivery systems [73].

Some research concerning the function of pH-responsive hydrogel confirmed these properties on cardiac tissue engineering after MI. pH-switchable supramolecular hydrogel has self-healing properties and delivered growth factors to the MI heart with a reduction in infarct scars in MI pigs [73]. An injectable hydrogel that is both pH- and temperature-sensitive can effectively afford heart-targeted delivery of angiogenic growth factors in MI rats, increase angiogenesis, enhance blood flow, and improve cardiac function [69]. In an animal model of myocardial infarction, growth factors are delivered from the hydrogel, resulting in reduced scarring, increased microvessel density, improved regional blood flow, and improved cardiovascular function [69,73]. pH- and temperature-responsive hydrogel-loaded oncostatin M can release an oncostatin M response to local pH and exert biological effects of oncostatin M in MI rats [66]. In addition to promoting angiogenesis, cardiomyocyte proliferation, and macrophage polarization, the composite hydrogel suppresses cardiomyocyte apoptosis and myocardial fibrosis, ultimately leading to improved cardiac function [66].

### 5.3. Ion-Sensitive Hydrogels

Ion-sensitive hydrogels have properties that electrical conductivity can be changed in different ionic conditions. Typically, a hydrogel is in a liquid state with fixed charges on the polymer backbone after synthesis, and transitions from solution state to gel with the interaction between charged ions and counterions in a specific electric field [74]. The metal ion- and thermal-responsive shape-morphing hydrogels actuate with good mechanical properties [75].

Salt- or temperature-induced cooperative swelling–shrinking properties control the speed, amplitude, and degree of bidirectional bending, as well as the angle at which it occurs and the degree at which it occurs [75]. The conductivity and contract ability of cardiomyocytes were significantly impaired after MI, which is challenging the electroconductive performance of on-responsive hydrogels. Researchers found that a hydrogel with ion-responsive properties can facilitate the assembly of cardiomyocytes into functional synctiums thanks to its high flexibility, good electrical conductivity, and tunable mechanical properties [76]. Ion-sensitive hydrogels have several advantages, including their ionic nature and biocompatibility, their ability to absorb and deliver negatively charged agents [53], and their ability to release drugs effectively in the gastrointestinal tract, which affects drug release both based on pH and ionic environments [77]. Most ion-responsive hydrogels that exhibit excellent positive charge density adhere to tissue surfaces with negative charge densities [77] and enable the loading of small molecules, as well as the tunable release of macromolecules [78].

### 5.4. Hypoxia-Responsive Hydrogels

Hypoxia plays an essential role in the process of MI, which can accelerate cell death, tissue necrosis, the loss of membrane integrity, and cardiomyocyte apoptosis. Mitigating hypoxia and promoting cell survival has been recognized as a promising therapeutic strategy and is taken into consideration in hydrogel design [79]. Oxygen-generating hydrogels have been applied in the treatment or regeneration of infarcted myocardial tissue with the function of relieving metabolic stress, enhancing cell viability and reducing cell death [80]. In chronic hypoxic wounds, the oxygen-releasing hydrogel might promote human umbilical vein endothelial survival, migration, and tube formation [81]. So, oxygen release is essential for the development of hypoxia-responsive hydrogels.

Calcium peroxide (CPO) is an important material for oxygen-generating hydrogels, which can release hydrogen peroxide (H_2_O_2_) reacting with water. The H_2_O_2_ can release oxygen, undergoing a catalase reaction [82]. Finally, the end products of CPO are water, oxygen, and calcium hydroxide. Calcium hydroxide has detrimental effects on cell growth. As well as causing acid-base imbalance, CPO also inhibits the regeneration of cells. By introducing Vitamin C, the out-of-balance acid-base challenge was successfully overcome and excessive ROS production was reduced [74]. Hydrogel crosslinked with CPO incorporated into a dynamic horseradish peroxidase (HRP) matrix can control the production of H_2_O_2_ [83]. By relieving metabolic stress, oxygen-generating hydrogels can enhance cardiac cell survival and performance [80]. Hydrogels that release oxygen can also be made from polyurethane polymeric materials (PUAO) incorporated with CPO. These hydrogels can prevent free radicals and oxidative stress-induced cell death [82]. PUAO in hydrogels can prolong the release duration of oxygen and avoid hyperoxia, maintaining the appropriate redox balance [82,84].

An oxidative cross-linking reaction mediated by calcium peroxide can produce hydrogel networks in situ from thiolated gelatin (GtnSH) in hyperbaric conditions. Angiogenesis-induced tissue infiltration and vascular recruitment are promoted by this hydrogel in wound healing [85]. By polymerizing NIPAAm, acrylate-oligopeptides (AOLA), 2-hydroxyethyl methacrylate (HEMA), poly (ethylene glycol)-perfluorooctane (MAPEGPFC), and reversible addition-fragmentation chain transfer (RAFT) were used to synthesize fast gelation hydrogels with high oxygen retention [86]. As cell carriers for cell transplantation into ischemic tissues, these high-oxygen hydrogels can enhance cardiomyocyte survival [86].

### 5.5. ROS-Responsive Hydrogels

As a consequence of cardiomyocytes having the highest number of mitochondria, they suffer from impaired mitochondrial function and impaired ATP metabolism following acute MI [87]. ROS plays a key role in several biological functions of the heart after MI, including cell communication and signal transduction, inflammation regulation, cell proliferation, and apoptosis [88]. When ROS are overproduced, they cause oxidative stress and inflammation, causing irreversible damage to the myocardium, and necrosis, inflammation, and fibrosis result after MI [89]. ROS, including superoxide anions, H_2_O_2_, and hydroxyl radicals in the post-MI microenvironments, have been targeted for therapeutic interventions and scavenger the ROS levels in the heart is effective in treatments after MI [90,91].

Research confirmed that ROS-responsive hydrogels with antioxidant properties can ameliorate excess ROS signaling and suppress oxidative stress injury in cardiomyocytes after MI [92]. Hydrogels that respond to ROS have been developed for localized delivery of liposomes containing drugs, which scavenge excess ROS and improve mitochondrial dysfunction in damaged cardiomyocytes [93].

Through the H-abstraction reaction, the glutathione (GSH) conjugated chitosan chloride (CSCl) chain can weaken hydrogen bonds and scavenge ROS [90]. In addition, CSCl-GSH from the amino group has formed stable macromolecule radicals and the sulfhydryl group has formed stable free radicals [91].

The CSCl-GSH hydrogel can effectively reduce intracellular ROS production, scavenge ROS, suppress oxidative stress, enhance the adhesion and survival of cardiomyocytes, and diminish cardiomyocyte apoptosis with excellent biocompatibility at the condition of high ROS after MI [92]. An injectable chitosan-based hydrogel might release nitric oxide (NO) when reacting with ROS and modulate the equilibrium between NO and ROS. It promotes the repair of the heart and improves cardiac function after ischemia/reperfusion injury by effectively attenuating cardiac damage and adverse cardiac remodeling [94]. Another research developed NO-releasing ROS scavenging hydrogels containing l-arginine and 4-amino-2,2,6,6-tetramethylpiperidine-1-oxyl (TEMPO) groups, which can regulate the NO sustained release and redox equilibrium, decrease the infarction size, and improve the heart function by enhancing angiogenesis [95]. A thermally responsive hydrogel, derived from antioxidant tetraaniline conjugated with NIPAAm, had favorable biocompatibility, excellent electrical and antioxidant activity, showed a time-dependent ROS scavenging effect, and supported cell proliferation and differentiation from ROS in vitro [96]. A TEMPO-containing injectable hydrogel significantly reduced acute infarction/reperfusion injury and improved cardiac function by extending retention time for ROS scavenging and reducing inflammation in rats [97].

The ROS-responsive hydrogels aim to reduce intracellular ROS production, scavenge ROS, ameliorate excess ROS signaling, and suppress the oxidative stress in the cardiac microenvironment to reduce the damage after MI.

## 6. Approaches of Hydrogel-Based Cardiac Regeneration

**Figure 3:**
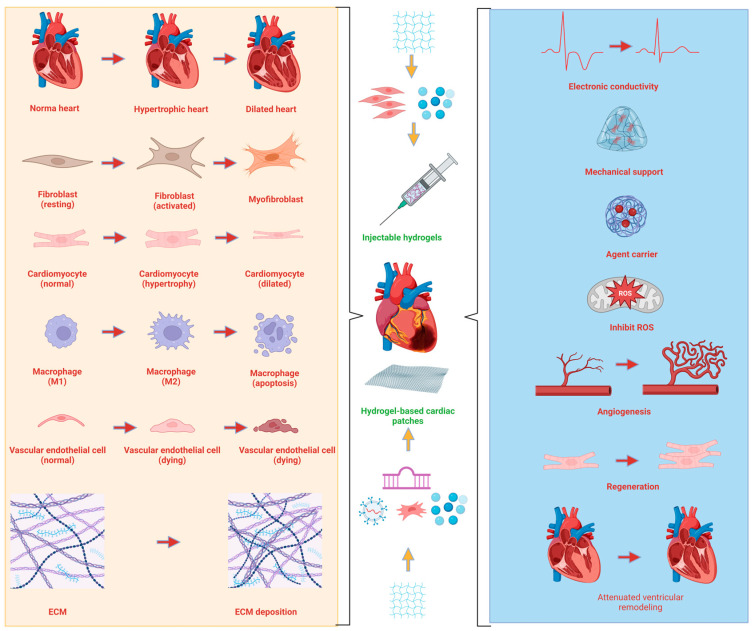
The pathophysiological changes of myocardial infarction and the applications of hydrogels in cardiac repair and regeneration for MI. Created with BioRender.com.

There are two main approaches to cardiac regeneration based on hydrogels: injectable hydrogels and hydrogel-based cardiac patches [98]. Injectable hydrogels are used to deliver bioactive compounds and cells to the myocardial wall for in situ repair and regeneration, and hydrogel-based cardiac patches provide mechanical support to the myocardial wall, inhibiting ventricular enlargement passively [99]. An injectable hydrogel patch is an emerging approach that hybrid injectable hydrogels and cardiac patches facilitate cardiac neovascularization and regeneration [48,98]. In order to repair damaged myocardium, a hydrogel patch must be surgically sutured to the epicardial surface. A growing interest has been generated in injectable hydrogels for the treatment of MI due to their ability to precisely enter and fill inaccessible tissue sites and pathological sites to repair damage (Figure 3).

### 6.1. Injectable Hydrogels

Generally, injectable hydrogels are liquid systems in vitro, but when they reach the myocardium, they transform into semisolid gels under specific physiological conditions. Microenvironment stimulation contributes to hydrogel gelation, retention, and controlled release of bioactive agents, including temperature, pH, ion, ROS, hypoxia electrical stimulation, and ischemia, which makes the hydrogel into a stimuli-responsive hydrogel and broadens its application scenarios. To prevent an immune response and similarity with native tissue, hydrogels must be biocompatible, biodegradable, and absorbable by the surrounding environment. It is important to take into account the contractility and elasticity of the material for coping with periodic contractions and relaxations [100]. Furthermore, mechanical stiffness, biochemical and physical suitability of the microenvironment and post-implantation retention time of bioactive compounds should all be considered [101].

As an injectable biomaterial, these factors should be taken into consideration for the design of injectable hydrogels, the bioactive compound encapsulated in the hydrogel in vivo, administration route and time of hydrogels, the required stimuli for release of bioactive compound, gelation time, the controlled release of bioactive compounds, and the clearing of hydrogels [6].

Typically, injectable hydrogels are administered intramyocardially, intracoronary, or intravenously with the help of a cardiac catheter. As a minimally invasive administration method, injection decreases healing time, facilitates the encapsulation of cells and biomolecules, and minimizes infection risk.

A key function of injectable hydrogels is to promote angiogenesis, which is a crucial element of cardiac regeneration. Injecting hydrogels into the superficial myocardium promotes neoangiogenesis and attenuates pathologic remodeling [102]. There are several advantages to engineering vascular analogs and vascularized hearts with injectable hydrogels. As a result of their mechanical properties, hydrogels provide adequate hydraulic pressure for blood flow while maintaining a perfusive vascular architecture. Furthermore, hydrogels provide blood vessels with an entrance and promote vascularization in vivo via their interconnected porosity. The biological properties of hydrogels include the ability to attach to and communicate with cells, thus helping in the vascularization processes.

As well as increasing the thickness of the ventricular wall, an injectable hydrogel enhances the geometry of the left ventricle, supports myocardial tissue, and promotes myocardial tissue regeneration [103]. Meanwhile, an injectable hydrogel could relieve infarct expansion and left ventricular dilation by reducing the pressure on the ventricular wall. Furthermore, an injectable hydrogel significantly improves cardiac function and has good safety and therapeutic effectiveness [104].

Restoration of cardiac function has been shown to be accompanied by an increase in healing, cardiomyocyte survival, and a decrease in pathological remodeling of the myocardium [105]. Cell transplants using hydrogels can be performed with minimal invasion of the human body [106]. Myocardium remodeling and infarct dilation after MI can be reversed with injectable self-healing conductive hydrogels. In addition to reducing the electrical resistivity of myocardial fibrous tissue, the hydrogels can also enhance the conduction velocity of myocardial tissue, which contributes to the restoration and maintenance of electrical conductivity after MI [107].

Research confirmed that injectable hydrogels with preliminary feasibility have the potential as endogenous tissue repair for cardiac regeneration, mainly due to their ability to improve cell adhesion and proliferation, and are regarded as the candidates of translatable agent carriers.

The mechanisms of injectable hydrogels in cardiac regeneration are still unclear, and these factors involve the role of cardiac regeneration after MI. The injectable hydrogel can prevent inflammatory response, improve local neovascularization, rescue at-risk cardiac cells via the loaded miRNA, rescue the ischemic myocardium through promoting angiogenesis by the delivered proangiogenic factors, and improve and optimize the microenvironment in the damaged heart tissue.

Overall, the type and number of encapsulated compositions in hydrogels, composition consistency, biocompatibility, gelation, retention, controlled release of bioactive agents, and biodegradation can influence the function and potential harm. Different stimuli types make the application of injectable hydrogels more complicated; thus, additional work is needed to resolve this issue.

### 6.2. Hydrogel-Based Cardiac Patches

Injectable hydrogels have demonstrated promise as an alternative biotherapeutic for cardiac regeneration following MI. Despite injection systems’ ability to prevent ventricular dilatation and enhance myocardial repair, there are still significant limitations. These include poor mechanical properties, potential immunogenicity, being washed away rapidly from the beating heart, and low retention of cells or drugs, which limit their therapeutic efficacy. These limitations can be overcome by hydrogen-based cardiac patches, which have received attention from various medical fields. The hydrogen-based cardiac patches enable drug retention with enhanced loading capability, bioavailability, biodistribution, stimulation responsiveness, controlled on-demand drug release, and multifunctional therapeutic demands, thus improving efficacy and reducing systemic toxicity. Due to electrically conductive and nanofibrous networks, optimal adhesion, organization, and communication between cells are achieved. Three-dimensional biohybrid actuators formed from patches exhibit controllable linear contraction and extension, pumping, and swimming behaviors. The degradation including the degradation time and the nanotubes after the degradation is a critical property of hydrogel-based cardiac patches. The cardiac patch must persist for the repair process, and research confirmed the hydrogel did not significantly degrade over 21 days when tested in vitro [30]. Long-term retention of hydrogel base patches may benefit the long-term support of the ventricle, while long time retention of foreign patches may increase inflammation or calcification. The degradation also allows hydrogels to provide bioactive effects and then degrade after they confer regenerative effects. The nanotubes after the degradation of the hydrogel still need to investigate thoroughly.

Hydrogel-based cardiac patches, which are incorporated with carbon nanotubes as electrical nano-bridges and electrical coupling, can achieve synchronous beating between cardiomyocytes as native myocardial tissue and can be used for various heart defects repair [33] and damaged myocardium regeneration [108,109]. Hydrogel’s viscoelasticity allows it to adapt to the dynamic surroundings during heartbeats, which may facilitate the construction of a unique heart. However, the viscoelasticity stages affect cardiac output [110]. Furthermore, hydrogel’s biocompatible properties facilitate cell proliferation and minimally invasive implantation procedures [111]. The electroactivity of patches is another important consideration to re-establish electrical coupling with infarcted myocardium and improve defective electrophysiology [112,113], which is crucial to preventing asynchronous contractions of the ventricular chambers and malignant arrhythmias [114,115]. Acid doping of polymers, which gives them conductive properties, can also reduce their biocompatibility [116], which should be taken into consideration. Mechanical–electric coupling behaviors were also modulated by myocardial patch interface binding performance [117].

Using hydrogel patches with electronic conductivity, cardiomyocytes in a rat MI model showed attenuated remodeling, improved contractility, and restored heart function [6]. A hydrogel-based cardiac patch blending decellularized heart matrix enhanced the cell survival rate and neonatal rat ventricular myocyte retention, improving contractile stress and electrophysiological function [118]. The studies on hydrogel-based cardiac patches in cardiac regeneration after MI are listed in Table 1.

Research in rats with MI showed that hydrogel-based cardiomyocyte-incorporated cardiac patches with good electronic conductivity attenuated left ventricular remodeling, improved contractile activity, and restored heart function [121]. The report confirmed cardiac patch on the heart surface can generate the greatest cardiac retention [122,123].

While hydrogel-based patches provide injectable viscoelasticity and myocardial-like toughness, their mechanical strength is low [124,125]. Moreover, the patch may leak into the thoracic cavity, which can adhere to the thoracic wall [126]. Most cardiac patches are suture-based, which is difficult to suture on the infarcted area and incur mechanical hazards. Therefore, biomaterial-based paintable and adhesive patches can be used to apply hydrogel-based cardiac patches without sutures.

In order to provide a strong wet adhesive property, conductive ions, and mechanical stability, a self-polymerized paintable hydrogel-based patch was developed [117]. Recently, the adhesive hydrogel cardiac patches afforded mechanical support, a regulatory effect on the microenvironment, which reduces ventricular wall stress, decreases the left ventricular cavity size, and restores the left ventricular shape [120]. A special type of hydrogel patch, a hydrogel-based exosome patch, has been applied in cardiac tissue engineering. A synthetic or natural patch was used to load exosomes and generate higher cardiac retention than intramyocardial injection [48,127]. Hydrogel patches loaded with MSC-derived exosomes without traditional implantation of cardiac patches typically require open-chest surgery [48]. Exosomes hybridized hydrogel patches have been proven to have wide applications in several conditions, such as reducing SCI-induced inflammation and glial scarring in spinal cord injury therapy [128], promoting cell migration, angiogenesis by slowly releasing exosomes in the deep layer of the skin in diabetic wound healing [129], and promoting cartilage defect repair and articular cartilage regeneration [129]. A hydrogel-based exosome patch exhibits great potential in the regeneration medical research and needs more research to explore the therapeutic function in the cardiac regeneration of MI.

The mechanical–electrical interactions of hydrogel patches with myocardium remain unclear, despite some hydrogel patches showing good therapeutic efficacy. Hydrogel’s mechanical properties, for example, should be explored to determine how they affect ventricular dilation and stroke volume, or how they affect the reconstruction of electrophysiological propagation. It is, therefore, imperative to develop hydrogel patches that can communicate mechanically and electrically with infarcted myocardium [98].

### 6.3. Potential Application of Hydrogels in Cardiac Repair and Regeneration after MI

As an important biomaterial, hydrogel shows exceptional promise in a wide range of applications including drug/gene delivery carriers [38], sensors [39], and scaffolds [40] in disease treatment, wound dressing [41], and tissue engineering [42], even in emerging areas such as wearable devices [43]. These applications of multidisciplinary and multidisciplinary techniques benefit the discovery and future development of cardiac repair and regeneration after MI. Hydrogels may bring new opportunities for the development of vascular grafts and nanocarriers, which may accrue benefits to the classical treatments of MI, including pharmacological treatment and revascularization strategies. The potential applications and the advantages and challenges are shown in Table 2.

## 7. Summary

Overall, the use of hydrogels in cardiac repair and regeneration after MI holds great promise. Hydrogel properties, such as bioactivity, biocompatibility, and biodegradability, improve cardiac regeneration and restore cardiac function in MI. Due to its rapid and repeated contraction, high conductivity, and high blood circulation, the heart has a lower colonization resistance and a high clearance from the blood, which limits the application scenarios of biomaterial-based therapy in MI. Hydrogels are ideal biomaterials for cardiac implants and agent delivery systems due to their high binding affinity, swelling ability, and absorption ability. Enhanced colonization resistance can be achieved through the cross-linked spatial structure of hydrogels, extending their half-life in vivo and preventing rapid degradation. Meanwhile, the hydrogel can enhance electrical conductivity, which is beneficial to the contraction. A hydrogel, whose physiochemical structure can be modified in response to external and intrinsic stimuli, such as temperature, pH, iron, hypoxia, and ROS, can be used as direct implantation onto the left ventricle with a controlled drug release system to improve the post-ischemic environment with angiogenic factors, to provide structural support to the injured region, and to enhance conductivity.

Hydrogel-based cardiac patches and injectable hydrogels are the most commonly used applications in cardiac regeneration medicine. With injectable hydrogels, bioactive compounds and cells can be delivered in situ, promoting in situ repair and regeneration, while hydrogel-based cardiac patches reduce myocardial wall stress, which passively inhibits ventricular expansion. The physiochemical structure rearrangements of intelligent hydrogels in response to intrinsic and extrinsic stimuli improved the maneuverability of hydrogels in cardiac research. Different stimulus types make the application of injectable hydrogels more complicated. Hydrogel-based cardiac patches work as mechanically supportive biomaterials. The advantages of intelligent hydrogels broaden the applications, enrich the regulation methods, and enhance the controllability of injectable hydrogels and hydrogel-based cardiac patches.

Hydrogels provide structural support to the injured area and are used to deliver therapeutic agents with controlled release. Hydrogels are synergistic with encapsulated bioactive agents. Therefore, both must act simultaneously in order for the effects of cardiac regeneration to be observed, which makes the mechanism complex and urgently needs clarification. The mechanism of hydrogels in cardiac regeneration medicine may involve reducing inflammation, limiting fibrosis, and enhancing angiogenesis. Further studies concerning hydrogel-based therapies are needed to understand their mechanisms.

Hydrogels must overcome certain challenges to be used clinically for cardiac regeneration. A majority of hydrogel research is performed on animals, and the immunogenic potential of hydrogels is completely unlocked, especially in vivo. From the bench to the bedside, hydrogels can still be improved. In addition, hydrogels can reduce the electroconductive ability of the heart, as hydrogels are electroconductive materials. Research confirmed that none of the hydrogels satisfy all the requirements for successful in vivo use. Hydrogel implantation procedures have dramatically reduced the risk of injury, but minimally invasive methods are still to be expected, especially for hydrogel-based cardiac patches. Electrical conductivity is influenced by the cardiac microenvironment, which is affected by the implantation of hydrogel patches. For patients with mild or moderate heart failure, open-heart surgery is not a feasible option. In the future, minimally invasive approaches and reliable delivery systems, such as injectable hydrogels, paintable patches, and adhesive patches without sutures, may offer promise. Taking these hydrogels from the lab to the clinic is challenging.

In cardiac regeneration medicine, clinical trials and commercial products are limited. Biomaterials, biochemistry, and biological actives, such as intelligent hydrogels and hydrogel-based exosome patches, which may serve as an effective treatment for MI in the future, are still under development. Further investigation of clinical feasibility is warranted. We can anticipate hydrogels having immense translational potential for cardiac regeneration in the future.

In conclusion, hydrogels hold great promise in cardiac repair and regeneration after MI. Intelligent hydrogels make structural rearrangements by physiochemical alterations in the surrounding medium, which broaden the application scenarios of hydrogels in cardiac tissue engineering. Injectable hydrogels are agents’ carriers of bioactive compounds and cells, while hydrogel-based cardiac patches reduce myocardial wall stress as mechanically supportive biomaterials. The challenges and opportunities coexist in cardiac tissue engineering. Further investigation of clinical feasibility is warranted. We can anticipate hydrogels having immense translational potential for cardiac repair and regeneration shortly.

## Figures and Tables

**Table 1:** The studies on hydrogel-based cardiac patches in cardiac repair and regeneration after MI in recent years.

Author/Year	Animal	Patch Properties	Hydrogel Composition	Heart Function Tests	Biological Properties	Bioactivity
Yu/2022 [98]	Rat heart	Intrapericardial injectable mechanical−electrical coupled hydrogel patch	Hydrogel patch combined with adipose-derived stem cells	Hemodynamics, electrophysiology, echocardiography. Electrocardiography, histology, and immunofluorescence	Enhanced hydrophilicity and the flexibility of hydrogel preparation, modulated the conductive network, conductivity maintenance, rapid preparation and easy injection, tailored to the shape and dynamic characteristics of the pericardial cavity	Effectively inhibited malignant ventricular fibrosis, dilatation, and thinning, promoted revascularization in the infarcted region and assisted electrical conduction and synchronous pulsation functions
Cheng/2022 [48]	Rat and pig hearts	Hydrogel-based exosome-derived from MSC patch	Exosomes isolated from MSCs	Echocardiographic, Masson’s trichrome staining, and wheat germ agglutinin staining	Protected the heart from adverse remodeling, reduced the LV chamber size, improved LV wall thickness, and reduced interstitial fibrosis	Protected cardiomyocytes from hypertrophy, promoted cardiac cell proliferation, and reduced cardiac cell apoptosis
Rodness/2016 [119]	Rat heart	VEGF-loaded hydrogel-based microsphere patch	Hydrogel patch loaded VEGF	Masson’s trichrome staining, α-SMA staining, gsisolectin b4 staining, and VEGF staining	An average diameter of 3.2μm, nonporous, smooth dimpled surface, and prolonged releasement	Patches promote the sustained release of bioactive VEGF and augmented LV function by promoting angiogenesis
Wu/2023 [120]	Rat heart	Wet adhesive hydrogel cardiac patch loaded with anti-oxidative and autophagy-regulating molecule capsules and MSCs	Hp-β-cd and resveratrol synthesized hydrogel integrated with antioxidant and autophagy bioactivities	Echocardiographic, histological evaluation, and immunofluorescence staining	The scavenging ability of the hydrogel cardiac patch came from the loaded anti-oxidative agents	Hp-β-cd protected cardiomyocytes via the promotion of autophagy, the reduction of oxidative stress damage in cardiomyocytes, and the restoration of mitochondrial function
Wang/2021 [112]	Rat and minipig hearts	Injectable and conductive cardiac patches	Patches seeded with rat cardiomyocytes and patches incorporating cardiomyocytes differentiated from human pluripotent stem cells	Echocardiography, conductivity assessment, epicardial activation mapping, histology, and immunofluorescence evaluation	Maintained a constant storage modulus without any mechanical fatigue or failure	Functional repair after 4 weeks, as indicated by increases in fractional shortening, the ejection fraction, and by a decrease in the infarcted area

MSC, mesenchymal stem cell; LV, left ventricle; VEGF, vascular endothelial growth factor; α-SMA, α-smooth muscle actin.

**Table 2:** The potential applications and the advantages and challenges of hydrogels in cardiac repair and regeneration after MI.

	Applications/Potential Applications	Advantages	Challenges
Injectable hydrogels	Administered intramyocardially, intracoronary, or intravenously as the candidates of translatable agent carriers for cardiac repair and regeneration	Minimally invasive procedure, acceptable physicochemical and mechanical properties, provides a supporting matrix to protect the encapsulated cells/agents	Low retention of cells and drugs, hydrogels hard to adhere to heart surface, poor mechanical properties, potential immunogenicity, and being washed away rapidly from the beating heart
Cardiac patches	Surgically sutured to the epicardial surface	Improving efficacy and reducing systemic toxicity, providing mechanical support, improving the interaction between the cardiac patch and the host myocardium, enhancing retention, and promoting the controlled release of bioactive agents	Surgical trauma impairs the contraction relaxation and electronic conductivity, and hard to enter the damaged tissue
Nanocarriers	Delivery carriers for cells, proteins, drugs, and nucleic acids	Improve the survival and proliferation of cardiomyocytes and stem cells, and ensure a sustained release at the target sites, thereby enhancing the therapeutic efficacy and reducing the systemic side effects	The uncontrolled balance between biodegradation and mechanical strength
Vascular grafts	Tissue-engineered vascular grafts supply blood directly to the infarcted tissue apart from cardiac regeneration	Degradable, modifiable biocompatibility, nontoxic, and excessively nonimmunogenic	Enough mechanical properties in the beating heart with high blood pressure, thrombosis risk, and resistible with long-term complications

## Data Availability

Data sharing is not applicable.
